# Assessing patients’ acceptance of their medication to reveal unmet needs: results from a large multi-diseases study using a patient online community

**DOI:** 10.1186/s12955-018-0962-3

**Published:** 2018-07-06

**Authors:** Jérémy Lambert, Michael Chekroun, Hélène Gilet, Catherine Acquadro, Benoit Arnould

**Affiliations:** 1 Mapi, Patient-Centered Outcomes, 27 rue de la Villette, 69003 Lyon, France; 2 Carenity, 1 rue de Stockholm, 75008 Paris, France; 3 Mapi, Thought Leadership, 27 rue de la Villette, 69003 Lyon, France; 4 Now at IQVIA, 151-161 Boulevard Victor Hugo, 93400 Saint Ouen, France

**Keywords:** Acceptance, Questionnaire, Chronic disease, Observational study, Tolerability, Risk/benefit assessment, Convenience

## Abstract

**Background:**

Patients with chronic conditions are required to take long-term treatments for their disease itself or to prevent any potential health risks. Measuring patient acceptance of their medication should help to better understand and predict patients’ behavior toward treatment. This study aimed to describe the level of patient acceptance toward various long-term treatments in real life using an online patient community.

**Methods:**

This was an observational, cross-sectional study conducted through the French Carenity platform. All Carenity patient members were invited to complete an online questionnaire including the 25-item ACCEptance by the Patients of their Treatment (ACCEPT©) questionnaire. ACCEPT© measures patient acceptance toward their medication and includes one general acceptance dimension (Acceptance/General) and six treatment-attribute specific dimensions (scores 0–100; lowest to highest acceptance): Acceptance/Medication Inconvenience, Acceptance/Long-term Treatment, Acceptance/Regimen Constraints, Acceptance/Side effects, Acceptance/Effectiveness, and Acceptance/Numerous Medications. Patients included in the analysis were treated adults experiencing any chronic diseases and who responded to at least one ACCEPT© item.

**Results:**

Among the 4193 patients included in the analysis, more than 270 chronic diseases were represented, amidst which 19 included more than 30 patients. Mean ACCEPT© Acceptance/General score for those 19 diseases were 61.2 (SD = 31.9) for type 1 diabetes, 59.8 (SD = 32.3) for asthma, 56.3 (SD = 34.3) for hypertension, 52.0 (SD = 32.2) for chronic obstructive pulmonary disease, 51.7 (SD = 27.0) for epilepsy, 50.1 (SD = 33.1) for bipolar disorder, 49.9 (SD = 33.1) for type 2 diabetes, 48.6 (SD = 31.6) for multiple sclerosis, 46.1 (SD = 34.5) for Crohn’s disease/ulcerative colitis, 44.3 (SD = 31.5) for depression, 42.8 (SD = 31.5) for lupus, 42.3 (SD = 33.0) for arthrosis, 41.8 (SD = 32.6) for Parkinson’s disease, 40.5 (SD = 32.2) for rheumatoid arthritis, 38.6 (SD = 31.7) for breast cancer, 36.4 (SD = 36.4) for myocardial infarction, 35.8 (SD = 32.0) for ankylosing spondylitis, 34.1 (SD = 32.3) for psoriasis, and 33.7 (SD = 31.7) for fibromyalgia.

**Conclusions:**

This first of its kind study enabled ACCEPT© data to be collected in real life for a variety of chronic diseases. These data may help in evaluating and interpreting levels of acceptance in future studies and provide valuable insights about patient priorities and current unmet needs.

## Background

Management of most chronic conditions requires patients to take long-term treatments as prescribed by their physicians, either to treat the disease itself or to prevent any potential health risks.

The balance between the benefits of a treatment, such as its therapeutic efficacy, and the perceived risks of this treatment, such as adverse events and treatment constraints, is the basis for risk-benefit assessment [1]. However, the perspective of the patient is not necessarily the same as that of health care providers or other stakeholders and their view of “acceptable risk” may differ from the view of others.

Lack of adherence (i.e. The extent to which the patient’s behavior matches agreed recommendations from the prescriber [2]) and persistence (i.e. the act of continuing the treatment for the prescribed duration) are major barriers to treatment efficiency. Poor adherence and persistence are not only important issues worldwide, jeopardizing a large part of the investments being made by drug developers and health care systems, but they also show that many patients, across many conditions and diseases, are not really convinced that their drug is worth taking as prescribed by their doctor [3, 4]. Patients’ behavior and attitude toward their treatment most probably result from a complex, yet not fully understood, evaluation of treatment benefits and risks by the patients themselves. Understanding the reasons why patients, in a specific context, do not adhere to their treatments, is thus of critical importance [5, 6].

Horne and colleagues developed the necessity-concerns framework in which factors influencing patients’ evaluation of their treatments fall within two categories: their perceptions of personal need for treatment (necessity beliefs) and their concerns about a range of potential adverse consequences [7–9]. The Beliefs in Medication Questionnaire (BMQ), which aims to quantify those necessity beliefs and concerns, has proven to be predictive of patients’ adherence in several chronic conditions [10, 11]. However, the necessity-concerns model does not determine when the balance is in favor of taking the treatment or not, in other words, whether the patients, based on their personal experiences, think that the medication is worth being taken or not. The emerging concept of acceptance aims at filling this gap by exploring how easy to accept treatment attributes are to patients, and what treatment attribute could be considered as a challenge to patients. While adherence is part of a person’s behavior, acceptance would be better placed in between the determinants and the resulting adherence [12]. One might indeed hypothesised that the more patients would accept the harms, risks, and constraints of their medication, given the benefit they experience or expect from it, the more they would be likely to be adherent.

Acceptance arose from specific grounded qualitative research conducted in patients experiencing a range of various chronic conditions and aiming at exploring treatment tolerability [12]. The authors defined acceptance as the results of the balance between benefits (advantages) and risks (disadvantages) of a treatment as rated by the patients based on their own personal experience of their treatment [12]. Patients who were initially asked “whether their treatment was good or not” for them were not able or willing to express their views on this question but they commented on the ease or the difficulty for them to accept taking their treatment. Thus, measuring patient acceptance of their medication should help to better understand and predict patients’ behavior toward treatment and identify unmet needs. The generic ACCEptance by the Patients of their Treatment (ACCEPT©) questionnaire has been developed to measure how patients perceive advantages and disadvantages of their long-term medications [12, 13].

The present study aimed to describe, for a variety of chronic diseases, the level of patients’ acceptance of their long-term treatments in real life using an online patient community and by administering the ACCEPT© questionnaire.

## Methods

### Study design

This was an observational, cross-sectional study conducted through the French Carenity platform from May to September 2014 to include adult patients, experiencing any chronic diseases and currently receiving a treatment for this disease.

Carenity is a global online patient community created in 2011 in which both patients and relatives of patients concerned by a chronic disease can share their experience as well as information, find basic tools for health follow-up and contribute to medical research by generating real-world patient insights through online surveys. At the time that research was conducted, a total of around 30,000 members, patients and relatives, were registered on the Carenity platform as of May 2014. In addition, the platform at that time was only available in French. Members of the Carenity platform are regularly invited to participate to the surveys on a voluntary basis. Participants do not receive any financial incentives for their participation.

In this study, all patients registered on the French Carenity platform were invited to complete an online questionnaire including questions about demographics (age, gender, occupational status, geographic location), clinical characteristics (chronic disease, date of diagnosis, current treatment, comorbidities), and the 25-item ACCEPT© questionnaire. No directly identifying data have been collected. Patients received up to four invitation reminders. Participation in the study was on a voluntary basis, and no incentive was given to patients agreeing to complete the questionnaire. In addition, patients had no constraints in completion of the overall online questionnaire: no obligatory responses, no limitations in completion time and no obligation to fully complete the questionnaire.

As members of the Carenity platform, patients involved in this study were informed as they registered on the platform, and have consented expressly and specifically to the collection, handling and keeping of their personal and health data. They were also informed before starting the survey of the purpose of the study. The procedures followed for this study were in accordance with the French Law 78–17 of 6 January 1978 and its later amendments, and in accordance with the 1964 Helsinki declaration and its later amendments.

### ACCEPT© questionnaire

The ACCEPT© questionnaire is a generic patient-reported outcome questionnaire specifically developed to assess patients’ acceptance of long-term medications. Its development and its psychometric validation included a literature review, pharmacist and patient interviews, and a specific validation study conducted with 189 patients recruited through pharmacists [12, 13]. The ACCEPT© questionnaire comprises seven independent dimensions: one on general acceptance (Acceptance/General) and six treatment-attribute specific, covering all specific attributes of drug: Acceptance/Medication Inconvenience, Acceptance/Long-term Treatment, Acceptance/Regimen Constraints, Acceptance/Numerous Medications, Acceptance/Side effects, and Acceptance/Effectiveness (Table 1). While Acceptance/Numerous Medications is a single-item dimension, all other are multi-item.

**Table 1:** Description of the ACCEPT© questionnaire

Domains	Items	Content
Acceptance/Medication Inconvenience	5	Preparation, mode of administration, form, storage conditions for journeys, discreet uptake
Acceptance/Long-term Treatment	3	Past and future durations, routine
Acceptance/Regimen Constraints	5	Remembering to take, time to collect from the pharmacy, remembering to bring with one self, always having on oneself, frequency of administration
Acceptance/Numerous Medications	1	Having numerous medications
Acceptance/Side effects	5	Presence of side effects, unpleasant side effects, disabling side effects, need for supplementary drugs due to side effects, risk of serious side effects
Acceptance/Effectiveness	3	Efficacy, preventive effect, time to efficacy
Acceptance/General	3	Advantages vs. disadvantages, acceptability, worth taking medication in the long-term

The Acceptance/General score is based on three items: balance between advantages and disadvantages, overall acceptance of the medication, and whether the medication is worth taking in the long-term. These three items are rated with Likert-like scales.

The response scale common to all items from treatment-attribute specific dimensions gives the patients three response choices: “Yes and I don’t find this easy to accept”; “Yes and I find this easy to accept”; “No” for patients not considering the subject addressed in the item to be an issue. Each item can be interpreted in isolation or grouped with the other items of same dimension to form an aggregated score. Scores based on categorical/ordinal data are linearly transformed to range from 0 to 100 for all multi-item dimensions with a higher score indicating greater acceptance.

### Data analysis

Descriptive statistics were applied to describe the characteristics of the patient population and the ACCEPT© scores. Diseases with at least 30 patients were analyzed individually, while other diseases were grouped together in a group called “other.”

Patients who were included in the population on which the present analyses were conducted were patient meeting the following inclusion criteria: aged 18 years and above, resident in France, diagnosed with a chronic disease and currently receiving a treatment for this disease, and had completed at least one item of the ACCEPT© questionnaire.

When missing data occurred within one dimension of the ACCEPT questionnaire, personal mean score (PMS) imputation was used as per the user manual for that questionnaire. No missing data were allowed for other variables in the electronic questionnaire.

All data processing and analyses were performed using SAS software for Windows version 9.2 (SAS Institute, Inc., Cary, NC, USA).

## Results

### Study population

**Fig. 1:**
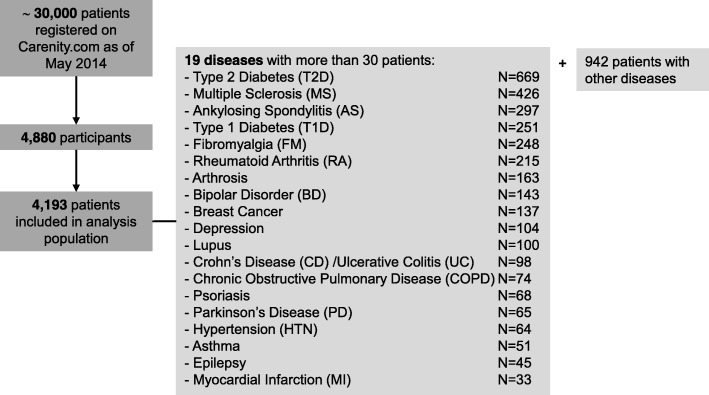
Patient population

Between May and September 2014, a total of 4880 patients responded to the invitation to participate in the study, and 4193 patients meeting the inclusion criteria were finally included in the analysis. More than 270 chronic diseases were represented, among which 19 included more than 30 patients (Fig. 1).

The demographic characteristics of the patient population are presented in Table 2. Median patient age was 54 years, ranging from 41.3 years for patients with Crohn’s disease/ulcerative colitis to 62.2 years for type 2 diabetes patients; the majority of patients were aged > 50 years (61%). Most of patientsin the overall population were female (71%; ranging from 47% for type 2 diabetes to 100% for breast cancer patients). The median time since diagnosis was 7 years in total, but varied widely across diseases, with the minimum time being 2 years for breast cancer and myocardial infarction and the maximum being 20 years for asthma patients. Forty-one percent of the patients had been diagnosed with their condition for at least 10 years. Overall, various patient profiles were observed according to the diseases.

**Table 2:** Demographic characteristics of the patient population (*N* = 4193)

	Total (N = 4193)
Age (years)	
Mean (SD)	52.8 (12.9)
Median (Q1 - Q3)	54.0 (44.0–62.5)
Min – Max	18.1–89.3
Age groups (%)	
< 40 years	17.5
40–49 years	21.9
50–59 years	28.7
≥ 60 years	31.9
Gender (%)	
Female	71.0
Male	29.0
Occupational status (%)	
Working	46.2
Unemployed	23.8
Retired	30.1
Time since diagnosis (years)	
Mean (SD)	10.7 (10.9)
Median (Q1 - Q3)	7.0 (3.0–15.0)
Min – Max	0.0–72.0
Groups by time since diagnosis (%)	
< 2 years	14.9
2–4 years	23.6
5–9 years	20.5
≥ 10 years	41.0

The majority of patients completed all 25 ACCEPT items (81% in total, with a minimum of 60% for Parkinson’s disease and a maximum of 92% for asthma). Regardless of the disease, patients missed to complete less than 1 item on average.

### General acceptance

**Fig. 2:**
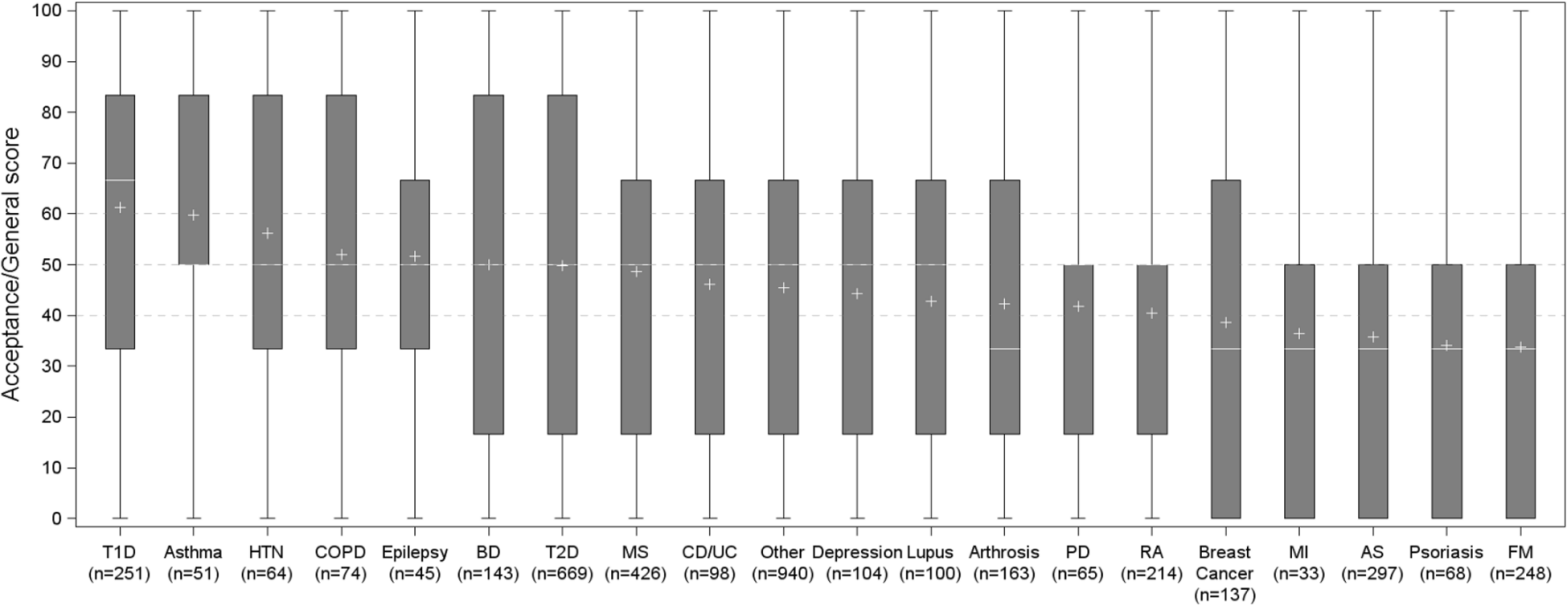
ACCEPT Acceptance/General score per disease

Figure 2 presents the mean Acceptance/General score, ranked from the highest mean (i.e., the best acceptance level observed) to the lowest mean (i.e., the worst acceptance level observed).

None of the diseases was observed to have an extremely high mean acceptance score; mean Acceptance/General score was below 65 for all diseases. The highest observed mean Acceptance/General score was for type 1 diabetes (61.2, SD = 31.9), while the lowest was for fibromyalgia (33.7, SD = 31.7). For all diseases, the minimum score observed was 0 and the maximum observed was 100, indicating that for each disease there were patients who completely accepted their treatment and patients who had major issues with accepting their treatment.

Purely based on the mean level of Acceptance/General score, with no pre-specified hypotheses, three groups of diseases were created post hoc. The first group of diseases was the one for which patients reported mean and median scores above 50 and for which the first quartile – the 25% of patients who report the lowest acceptance score – was not below 33. This group of diseases included type 1 diabetes, asthma, hypertension, chronic obstructive pulmonary disease and epilepsy. The second group of diseases comprised diseases for which mean and median scores were below 40, and at least 25% of patients reported the worst possible score, overall indicating major acceptance issues for these conditions. Breast cancer, myocardial infarction, ankylosing spondylitis, psoriasis and fibromyalgia were found in this group. The third group of diseases was the one for which patients reported a mean score between 40 and 50, a median score less than 50 and a first quartile at 16.7. For some of these conditions in this third group, a large variability was observed in the score reported by patients, as for type 2 diabetes with a very large interquartile range, while for some of the other conditions in this third group, scores reported by patients were quite consistent, as for Parkinson’s disease with a small interquartile range. The set of “other diseases” which included the remaining 940 patients with many different conditions and treatments was positioned exactly in the middle of all the specific diseases, with a mean score of 45.5.

### Treatment-attribute specific dimensions

Mean Acceptance/Medication Inconvenience scores ranged from 96.3 (SD = 7.8) for hypertension to 59.9 (SD = 29.3) for type 1 diabetes. All mean scores were above 70, expect for multiple sclerosis, ankylosing spondylitis and type 1 diabetes (Table 3).

**Table 3:** Description of ACCEPT© treatment attribute dimension scores, presented as mean (SD)

		Multi item dimensions					Single-item dimension
	N	Acceptance/Medication Inconvenience	Acceptance/Long-term Treatment	Acceptance/Regimen Constraints	Acceptance/Side effects	Acceptance/Effectiveness	Acceptance/Numerous Medications
Type 2 diabetes	669	80.0 (25.4)	56.0 (21.7)	67.0 (31.2)	71.4 (32.7)	67.0 (35.5)	36.8%
Multiple sclerosis	426	*69.7 (31.8)*	**57.8 (26.1)**	67.5 (28.0)	60.9 (33.1)	55.6 (38.2)	**20.7%**
Ankylosing spondylitis	297	*67.6 (28.6)*	50.4 (23.7)	63.1 (29.4)	*41.2 (33.5)*	*52.5 (38.4)*	37.7%
Type 1 diabetes	251	*59.9 (29.3)*	48.2 (24.0)	*52.9 (34.9)*	68.5 (34.8)	**79.8 (29.8)**	31.1%
Fibromyalgia	248	89.6 (16.8)	*47.4 (25.1)*	62.1 (32.2)	51.5 (34.0)	53.7 (35.9)	*52.4%*
Rheumatoid arthritis	215	75.6 (27.5)	48.6 (26.1)	67.5 (27.7)	*44.5 (34.9)*	57.3 (38.6)	*45.1%*
Arthrosis	163	**90.7 (16.1)**	**56.1 (23.5)**	**76.7 (26.6)**	70.7 (33.4)	59.8 (37.4)	25.8%
Bipolar disorder	143	90.4 (16.8)	49.9 (23.2)	62.1 (30.1)	46.1 (32.8)	67.0 (37.3)	39.9%
Breast cancer	137	86.5 (20.4)	53.5 (23.7)	71.1 (28.4)	*35.4 (29.8)*	*37.7 (34.6)*	27.7%
Depression	104	**91.3 (16.9)**	48.8 (23.4)	67.9 (30.3)	60.4 (33.1)	60.6 (38.7)	39.4%
Lupus	100	89.2 (16.5)	*46.3 (25.6)*	*60.2 (31.9)*	52.1 (34.6)	57.3 (35.3)	45.0%
Crohn’s disease/ Ulcerative colitis	98	72.9 (27.5)	50.8 (26.8)	67.2 (28.4)	53.6 (33.3)	62.2 (40.6)	23.5%
COPD	74	83.1 (21.8)	53.5 (22.7)	69.7 (29.7)	**77.8 (32.0)**	60.5 (36.0)	27.0%
Psoriasis	68	78.7 (22.4)	49.1 (28.0)	**71.8 (23.8)**	**74.6 (31.9)**	53.9 (37.6)	**11.8%**
Parkinson’s disease	65	88.5 (17.5)	*47.4 (25.9)*	*56.8 (33.8)*	52.1 (32.2)	54.9 (40.3)	36.9%
Hypertension	64	**96.3 (7.8)**	**61.3 (19.8)**	**73.4 (26.5)**	**81.4 (26.7)**	**77.6 (31.3)**	**20.3%**
Asthma	51	80.8 (22.7)	51.3 (17.6)	63.0 (28.1)	70.4 (36.3)	**75.2 (32.9)**	39.2%
Epilepsy	45	88.4 (21.0)	52.8 (22.9)	61.4 (28.9)	56.9 (28.8)	68.1 (37.4)	24.4%
Myocardial infarction	33	90.1 (18.8)	47.7 (26.3)	65.3 (33.1)	54.2 (32.4)	*51.0 (36.5)*	*54.5%*
Other diseases	942	84.9 (22.3)	53.7 (25.5)	67.8 (30.2)	57.8 (35.5)	60.7 (36.9)	30.0%
Total	4193	80.2 (25.7)	52.7 (24.6)	66.0 (30.4)	58.9 (35.3)	60.8 (37.4)	33.2%

*In bold, three diseases with best level of acceptance; in italics three diseases with worst level of acceptance*

ACCEPT© scores range from 0 to 100 for multi-item dimensions with a higher score indicating greater acceptance. The single item dimension “Acceptance/Numerous Medications” score is presented as the percentage of patients answering “I don’t find this easy to accept” from the original 3-point response scale common to all items

Mean scores for Acceptance/Long-term Treatment ranged from 46.3 (SD = 25.6) for lupus to 61.3 (SD = 19.8) for hypertension. All diseases showed scores < 66.7 and about half showed scores < 50.

Mean scores for Acceptance/Regimen Constraints were consistently in the upper part of the 0–100 range across all conditions (all mean scores > 50) and ranged from 52.9 (SD = 34.9) for type 1 diabetes to 76.7 (SD = 26.6) for arthrosis.

In contrast with the aforementioned dimension scores for which scores were quite consistent across diseases, the level of acceptance based on the Acceptance/Side effects and Acceptance/Effectiveness mean scores varied by more than 40 points between the condition with the highest level of acceptance and the one with the lowest. Hypertension had among the highest level of acceptance in both Acceptance/Side effects (81.4, SD = 26.7) and Acceptance/Effectiveness (77.6, SD = 31.3). In contrast, breast cancer had among the lowest level of acceptance in both Acceptance/Side effects (35.4, SD = 29.8) and Acceptance/Effectiveness (37.7, SD = 34.6).

Based on the single-item Acceptance/Numerous Medications, the majority of patients reported not being concerned or to find this easy to accept, with the exception of patients with myocardial infarction or fibromyalgia for which 54.5 and 52.4%, respectively, reported finding it not easy to accept.

## Discussion

This observational study is the first of its kind to collect data about patient acceptance of their treatment in real life for a wide variety of chronic diseases using a dedicated patient-reported outcome instrument.

The invitation to participate in the APTEO study was well received, with about 1 out of 6 of the community members having responded. Among the 4193 patients included in the analysis, more than 270 chronic diseases were represented, among which 19 included more than 30 patients. Mean ACCEPT© questionnaire Acceptance/General scores provided a contrasting picture across conditions, ranging from 61.2 (SD = 31.9) for type 1 diabetes to 33.7 (SD = 31.7) for fibromyalgia. The level of acceptance was quite consistent and stable within the different dimensions of ACCEPT© related to practical attributes, with high scores for Acceptance/Medication Inconvenience and lower score, but still on the upper half of the scale range for Acceptance/Long-term Treatment, Acceptance/Regimen Constraints, and Acceptance/Numerous Medications. However, findings were more contrasted for Acceptance/Side effects and Acceptance/Effectiveness scores, with more than 40-point differences observed between extreme mean scores.

The concepts of adherence and acceptance are not interchangeable. The Health Beliefs Model (HBM) presents the behavior of a person toward their health as a result of four major groups of determinants: the perceived severity of a condition, the perceived susceptibility to that condition, the benefits of taking a preventive action, and the barriers to taking that action [14–16]. Other variables, including demographic and individual characteristics, influence a person’s behavior [17, 18]. When developing ACCEPT©, we assumed that acceptance could be positioned in the HBM as a result of these determinants, and could capture the impact of these determinants on behavior. In other words, the more the patient would accept the harms, risks and constraints of their medication, given the benefit they experience or expect from it, the more they would be likely to be adherent and persistent. However, to date this hypothesized link between acceptance and adherence (or persistence) remains to be confirmed. A longitudinal study is currently being planned with the same cohort of participants to explore the correlation between the two concepts.

In the present study, in addition to the use of the concept of acceptance, the use of the Carenity online patient community may have contributed to avoiding various issues and biases. First of all, our study enabled the collection of data from more than 4000 patients within a couple of months. As demonstrated by others, social networks are an effective and rapid method of recruiting patients with various diseases [19, 20]. Access to patients was facilitated by the fact that social networks are attracting more patients to share their experiences and to be more involved in their health [21]. As any methods, there are limitations which have been discussed recently; for example, the data is spontaneously and sincerely provided by patients using a user name or alias, inability to reach certain communities [22]. Our study may still present a recruitment bias as we cannot exclude the possibility that we missed patients who do not have the skills and/or the access to online communities. However, in our study, almost one-third of all patients were aged > 60 years, with the oldest patients being aged almost 90 years. One may argue that patients sharing their experience or becoming members of online communities are those who tend to have negative experiences. In our study, the distribution of scores were going toward both the upper end and the lower end of the range depending on the disease. Still, for all diseases, we observed extreme values showing that in our sample there were both patients with the lowest possible general acceptance score, indicating that these patients had major issues with accepting their treatment and patients with the highest possible general acceptance score, indicating that these patients were completely accepted their treatment. Even if the mode of recruitment does not allow the sample to be considered a priori representative, there is evidence that – contrary to traditional recruitment through health care professionals – a large variety of experience is represented, showing very contrasted profiles across and within diseases.

Social networks offer the opportunity to collect information that is often not found in institutional records. In addition, the use of an online platform may have contributed to minimizing social desirability bias as participants who were involved were asked to report, in an anonymous manner, their behavior in real life outside the context of a clinical trial or of a study involving their doctors or other health care professionals. Patients self-reporting their level of adherence or acceptance directly to their physician may behave in a socially acceptable manner. It has been reported that this social desirability bias can lead to an inaccurate evaluation of adherence [23].

No direct comparison between the diseases were performed as this was not the objective of this study. Indeed, the objective was to present data on acceptance collected in real life for a variety of chronic diseases. For this reason, the statistical analysis performed were only descriptive and would be helpful to evaluate and interpret levels of acceptance in future studies. Moreover, the descriptive results obtained help describing the unmet needs of patients for each disease.

Our study shows for a series of diseases where treatment-related issues or unmet needs areprevalent. For example, patients with breast cancer, myocardial infarction, ankylosing spondylitis, psoriasis and fibromyalgia reported acceptance scores in terms of both effectiveness and side effects that were lower than other diseases (except psoriasis which had side effect acceptance score on the higher range of the scale). Our study highlights here the need to develop drugs that are more effective and associated with fewer side effects to increase patient acceptance of their treatment. This of course may not be an unexpected finding. However, even in disease areas for which patients did not report any acceptance-related issues with treatment safety and efficacy, there is room for improvement in other treatment attributes, such as mode of administration or treatment storage. This study gives clues where potential improvements could be needed and what aspects and priorities could be considered by the pharma industries when developing a new treatment to ensure it is well-accepted. For example, our findings suggest that for type 1 diabetes there is room for improvements in treatment convenience and constraints. Recent developments to reduce patient burden have been focused on releasing long-acting drugs with comparable safety and efficacy profiles, but reduced frequency of administration [24, 25]. An in-depth analysis at the item level could show exactly where there are potential issues.

The variations in the level of acceptance observed for each condition suggests that, in addition to the disease itself, other factors influence a patient’s acceptance. Disease stage can be such a factor, as well as choice of treatment (e.g., mode of administration, formulation), as several treatment options are frequently available to treat one disease. Studies have already demonstrated that repeated daily injection of insulin in patients with diabetes affects patients’ well-being and there is a search for alternative modes of administration [26, 27]. Further analysis per disease area is needed to investigate this level of detail regarding treatment and disease severity and how it affects the level of acceptance.

Our subset of participants (71% female, mean age 52.8 years) was very similar to the pool of contacted members of the Carenity platform (71% female, mean age 53.1 years). Slight differences were observed with regards to the proportion of contacted members with a disease versus the proportion of respondents with a disease. For example, among the contacted members, 10% had fibromyalgia, 9% type 2 diabetes and 5% type 1 diabetes, while in our subset, fibromyalgia patients represented 6%, type 2 diabetes patients 16% and type 1 diabetes patients 6%. Generalization of the findings from our study may be further limited because only French patients were included. We cannot exclude the possibility that patient management and health system may have an impact on patients’ behavior [28]. In addition, for some of the explored diseases, in particular epilepsy and myocardial infarction, the sample size was small, further limiting the possibility for generalizing the related findings. We are therefore planning to replicate this study in a larger sample including other European countries in which the Carenity platform has also been launched, in order to assess the generalizability of our findings. Comparing to the data of the French Health Insurance Information System for four chronic diseases (diabetes, multiple sclerosis, inflammatory bowel disease and Parkinson disease) an over-representation of female patients, aged from 25 to 54 years old was observed among the members of the French Carenity community [29].

## Conclusions

Overall, our first of its kind study enabled ACCEPT© data to be collected in real life for a variety of chronic diseases. Our data could be used as initial benchmark to start from to help in assessing and interpreting levels of acceptance in future studies. Furthermore, these data can contribute to provide indications on what the issues are that patients are facing with their treatment and that lead to lower acceptance, consequently identifying unmet needs and priorities for patients for future drug development. Future research is needed to confirm the findings in broader samples and an expanded geographic scope. The relationship between acceptance and adherence need to be explored.

## Abbreviations

ACCEPT: ACCEptance by the Patients of their Treatment
AS: Ankylosing spondylitis
BP: Bipolar disorder
CD: Crohn’s disease
COPD: Chronic obstructive pulmonary disease
FM: Fibromyalgia
HTN: Hypertension
MI: Myocardial infarction
MS: Multiple sclerosis
PD: Parkinson’s disease
RA: Rheumatoid arthritis
SD: Standard deviation
T1D: Type 1 diabetes
T2D: Type 2 diabetes
UC: Ulcerative colitis
